# Heterologous Expression and Characterization of a Novel Exo-Polygalacturonase from *Aspergillus fumigatus* Af293 and Its Application in Juice Extraction

**DOI:** 10.4014/jmb.2211.11003

**Published:** 2022-12-30

**Authors:** Chengwei Yang, Ting Zhang, Jing Zhu, Yunyi Wei, Furong Zhu, Zhong Cheng

**Affiliations:** 1 College of Food Science and Quality Engineering, Nanning University, Nanning, Guangxi 530200, P.R. China; 2 College of Life Science and Technology, Guangxi University, Nanning, Guangxi 530004, P.R. China

**Keywords:** Exo-polygalacturonase, *Aspergillus fumigatus*, acid stability, juice extraction

## Abstract

Exo-polygalacturonase (exo-PG) hydrolyzes pectin acids and liberates mono-galacturonate, which plays an important role in juice extraction, and has rarely been reported. Exo-PG (AfumExoPG28A) from *Aspergillus fumigatus* belongs to the glycoside hydrolase 28 family. In this study, its gene was cloned and the protein was expressed and secreted in *Pichia pastoris* with a maximal activity of 4.44 U/ml. The optimal temperature and pH of AfumExoPG28A were 55°C and 4.0, respectively. The enzyme exhibited activity over almost the entire acidic pH range (>20.0% activity at pH 2.5-6.5) and remained stable at pH 2.5–10.0 for 24 h. The *K*_m_ and *V*_max_ values of AfumExoPG28A were calculated by the substrate of polygalacturonic acid as 25.4 mg/ml and 23.6 U/mg, respectively. Addition of AfumExoPG28A (0.8 U/mg) increased the light transmittance and juice yield of plantain pulp by 11.7% and 9%, respectively. Combining AfumExoPG28A (0.8 U/mg) with an endo-PG (0.8 U/mg) from our laboratory, the enzymes increased the light transmittance and juice yield of plantain pulp by 45.7% and 10%, respectively. Thus, the enzyme’s potential value in juice production was revealed by the remarkable acidic properties and catalytic activity in fruit pulp.

## Introduction

Pectin is a kind of heteropolysaccharide composed of galacturonic acid linked by an α-1,4-glycosidic bond to form the main chain, and methanol and 12 monosaccharides are linked to the main chain [1]. Pectin is widely found in plant cell walls and membranes, along with cellulose and hemicellulose, which contribute to the toughness of plant tissues [2]. The pectinases are a group of multi-enzyme complexes capable of degrading pectin, and include polygalacturonase (PG), pectinesterase, and pectin/pectic acid lyase [3]. Pectinases have applications in a variety of industries, such as wine brewing [4], oil extraction [5], the paper and pulp industry [6], fruit and vegetable processing [7], coffee and tea fermentation [8], animal feed processing [9], and wastewater treatment [10].

PG belongs to the glycoside hydrolase 28 (GH28) family and is one of the most important pectinases due to its ability to cleave the α-1,4-glycosidic bond in the pectin backbone by (endo- or exo-) hydrolysis, resulting in high depolymerization efficiency [11]. In the pectinase family, exo-polygalacturonase (exo-PG) targets the terminal groups of the pectic molecule, leading to liberation of mono-galacturonate [12]. Acidic PG, mainly from filamentous fungi, can promote the release of pectin components by degrading fruit and vegetable cell walls, so it is widely used in fruit and vegetable juice extraction processes [13]. Due to the different pH values of various fruit pulps, the pH of any processed pulp should be matched to the pH stability range of the PG employed, which not only improves the production process (adjustment of juice pH) but also reduces the production cost [14]. Thus, the fruit juice industry seeks to discover PGs that are active and stable over a broad pH range.

*Aspergillus fumigatus* is a powerful producer of cell wall-degrading enzymes, such as β-glucosidase [15], phytase [16], and ligninase [17]. In this study, an exo-PG-encoding gene (AfumExoPG28A) from *A. fumigatus* Af293 was cloned and heterologously expressed in *Pichia pastoris*. The recombinant protein (AfumExoPG28A) was purified and characterized. The purified enzyme showed excellent acid stability and a broader acid activity range than most reported fungal counterparts. Moreover, AfumExoPG28A showed notable synergistic effect with endo-PG in the extraction of fruit juice. To the best of our knowledge, AfumExoPG28A is the first identified exo-PG from *A. fumigatus*, and the abovementioned properties suggest its potential value in the production of fruit juice.

## Materials and Methods

### Strains and Reagents

The vector pPIC9K (Invitrogen) and *P. pastoris* GS115 (Invitrogen, USA) were used for gene cloning and expression, respectively. The codon-optimized gene sequence of exo-PG (GenBank Accession No. 3512150) from *A. fumigatus* Af293 (GenBank Accession No. txid330879), was synthesized without the signal peptide-encoding region by Sango Biotech (China).

SacI restriction endonuclease was obtained from TaKaRa Bio Co., Ltd. (China). The endo-β-N-acetylglucosaminidase H (Endo H) was obtained from NEB (China). Yeast extract and peptone were purchased from Oxoid Limited (UK). D-galacturonic acid, PGA (de-esterified), citrus pectin (<26% esterified), and apple pectin (50–75%esterified) were purchased from Aladdin Reagent Co., Ltd. (USA). The TIANprep Mini Plasmid Kit was obtained from Tiangen Biotech Co., Ltd. (China). Other reagents were of analytical grade and commercially available.

### Media and Cultivation Conditions

*Escherichia coli* carrying the recombinant plasmid pPIC9K-*AfumExoPG28A* was cultured in Luria-Bertani (LB) medium (1% tryptone, 0.5% yeast extract, and 0.5% NaCl, w/v). *P. pastoris* carrying the exo-PG gene AfumExoPG28A was cultured in minimal dextrose (MD) medium (1.34% YNB, 4 × 10^−5^ g/l biotin, and 2% agar) or yeast extract peptone dextrose (YPD) medium (1% yeast extract, 2% peptone, and 2% glucose). To express the exo-PG gene, *P. pastoris* was cultured in buffered glycerol-complex (BMGY) medium (pH 6.0, 1% yeast extract, 2% peptone, 1% glycerol, 1.34% Yeast Nitrogen Base (YNB), 4 × 10^−5^ g/l biotin, and 0.1 M potassium phosphate buffer) and then transferred into buffered methanol-complex (BMMY) medium (pH 6.0, 1% yeast extract, 2%peptone, 1% methanol, 1.34% YNB, 4 × 10^−5^ g/l biotin, and 0.1 M potassium phosphate buffer).

### Construction of Recombinant Vector and Sequence Analysis

The gene was amplified by the primers [5´-GCGCGAATTCGCTCCTAACCAACCAATTCA-3´ (EcoRI was underlined) and 5´-ATATGCGGCCGCTTAGTGTGGAACGAAGGTAC-3´ (NotI was underlined) ], and the amplification procedure was followed according to a previous report [18]. Then, the gene was digested with EcoRI and NotI, and inserted into the vector pPIC9K.

The gene sequence of exo-PG (GenBank accession no. 3512150) from *A. fumigatus* Af293 (GenBank Accession No. txid330879) was obtained from NCBI (https://www.ncbi.nlm.nih.gov/). Based on the amino acid sequence of AfumExoPG28A from *A. fumigatus* Af293, the signal peptide of AfumExoPG28A (1–21 aa from the N-terminus) was confirmed by SignalP4.1 (http://www.cbs.dtu.dk/services/SignalP/). The ExPASy online server (http://web.expasy.org/protparam/) was used to calculate the hypothetical molecular weight of the protein. DNAMAN software was used to carry out the multiple sequence alignment.

### Transformation and Heterologous Expression

Recombinant plasmid pPIC9K-*AfumExoPG28A* was extracted using a plasmid extraction kit. Next, 1 μg of recombinant plasmid linearized by Sac I was transformed into *P. pastoris* GS115 through the LiCl method. After preliminary screening on MD plates, the positive transformants were selected by 2.5 mg/ml G418 on YPD plates and confirmed by PCR using the primers 5´AOX (5´-GACTGGTTCCAATTGACAAGC-3´) and 3´AOX (5´-GCAAATGGCATTCTGACATCC-3´) .

Next, in a 250-ml flask, one transformant was selected and inoculated into 50 ml BMGY liquid medium, cultured at 30°C and 200 rpm. When the OD_600_ reached 3.0, the cells were centrifuged at 2,000 ×*g* for 10 min at room temperature, and the supernatant was discarded. The cells were transferred to 25 ml BMMY liquid medium in a 250-ml flask and cultured at 30°C and 200 rpm. To induce the expression of AfumExoPG28A, 0.5% methanol was added in daily and the culture solution was sampled for enzyme production every 24 h. After 120 h of methanol induction, the culture solution was centrifuged at 4°C and 12,000 ×*g*. The supernatants were used as crude enzyme.

### Purification and Determination

The 100 ml culture solution of recombinant protein was concentrated with a molecular weight cut-off of 10 kDa using an ultrafiltration device (Ireland). Then, 20 mM Tris-HCl buffer (pH 7.0) was used to replace the culture solution, and the final solution was stored at 4°C until further analysis. The protein concentration was determined by the Bradford method [19]. The homogeneity of the protein was checked via SDS-PAGE (sodium dodecyl sulfate-polyacrylamide gel electrophoresis, using 5% stacking gel and 10% separating gel) [20]. The purified recombinant protein was deglycosylated by Endo H (20 μl reaction system: 20 μg protein, 3 μl enzyme dosage, and 2 μl 10 × GlycoBuffer 3 in ddH_2_O 37°C for 3 h) and then analyzed by SDS-PAGE.

### Enzyme Activity Assay

The activity of AfumExoPG28A was measured by the 3,5-dinitrosalicylic acid (DNS) method [21]. One unit of polygalacturonase activity is defined as the amount of enzyme required to release 1 mmol of D-galacturonic acid per minute [22].

### Determination of the Optimum Temperature and pH for AfumExoPG28A Activity and Stability

The optimal pH for AfumExoPG28A was determined at 50°C in the range of pH 2.5 to 7.0. The maximum enzyme activity was set as 100%, and enzyme activities at other pH values were normalized to this maximum value. The stability of AfumExoPG28A at different pH values was analyzed in different buffers (0.1 M citric acid-Na_2_HPO_4_ for pH 3.0–7.0, 0.1 M Tris-HCl for pH 7.0–9.0, and 0.1 M Gly-NaOH for pH 9.0–10.0) at 4°C for 24 h. The residual activities were determined after 15 min treatment at 50°C and at optimal pH.

The optimum temperature of AfumExoPG28A was analyzed at 25-80°C under optimal pH conditions. The maximum enzyme activity was defined as 100%, and other enzyme activities were calculated as relative values. To explore its thermostability, the enzyme solution was incubated at 45, 50, and 55°C for 1 h, and the residual activity was measured every 15 min.

### Determination of Kinetic Parameters and Substrate Specificity

To determine the kinetic parameters, different concentrations (0.20-1.0%) of PGA were used to measure the enzyme activity under optimal conditions (pH 4.0 and 55°C). The *K*_m_ and *V*_max_ values were calculated by the Lineweaver-Burk method. For the substrate specificity, 0.5% (w/v) PGA, 0.5% (w/v) citrus pectin, 0.5% (w/v) apple pectin, 0.5% (w/v) carboxymethyl cellulose, and 0.5% (w/v) xylan were used under optimal conditions. The enzyme activity of AfumExoPG28A with 0.5% (w/v) PGA was defined as 100%, and activities in other reactions were normalized to this value.

### Effects of Metal Ions on AfumExoPG28A Activity

The presence of metal ions may affect enzyme activity in many ways. To determine the effects of metal ions on enzyme activity, the effects of 1 or 2 mM of Zn^2+^, K^+^, Ca^2+^, Mg^2+^, Ba^2+^, Na^+^, Mn^2+^, Cu^2+^, Co^2+^, and Fe^2+^ on the catalytic activity of AfumExoPG28A were measured. The control value with no metal ions was defined as 100%, and activities in other reactions were normalized to this value.

### TLC Analysis of Hydrolysis Products of PGA by AfumExoPG28A

The purified samples of AfumExoPG28A and 0.5% PGA with 0.1 M citric acid-Na_2_HPO_4_ (pH 4.0) were incubated at 55°C for 0.25, 0.5, 1.0, 2.0, 8.0, and 12 h. The control sample did not contain AfumExoPG28A. The main hydrolysis products were analyzed by TLC to demonstrate the action mode of AfumExoPG28A using a previously reported method [23].

### Use of AfumExoPG28A to Extract Fresh Plantain Juice

Fresh and disease-free fruits (plantain) were purchased at a local market. Next, 500 g fruit and 500 ml double-distilled water were mixed and pulped. AfumExoPG28A (0.8 U/mg) was added to fresh pulp (25 g), and samples were incubated for 2 h at 55°C. Then, an endo-PG (0.8 U/mg) from our laboratory and AfumExoPG28A (0.8 U/mg) were mixed together in fruit pulp under the same conditions. The same amount of pulp was also incubated without enzyme as a control. Then, fruit pulp in the control and experimental groups was centrifuged at 3,214 ×*g* for 10 min at 25°C. The supernatants were measured to determine the pH, juice extraction rate (1), and light transmittance (A_660_) using previously reported methods [21].

$$\text{Juice extraction rate (\%) =}\frac{\text{clear juice weight of experimental group (g)}}{\text{weight of fruit pulp (g)}}$$

## Results and Discussion

### Gene Cloning, Expression, and Purification of Recombinant Exo-PG

**Fig. 1:**
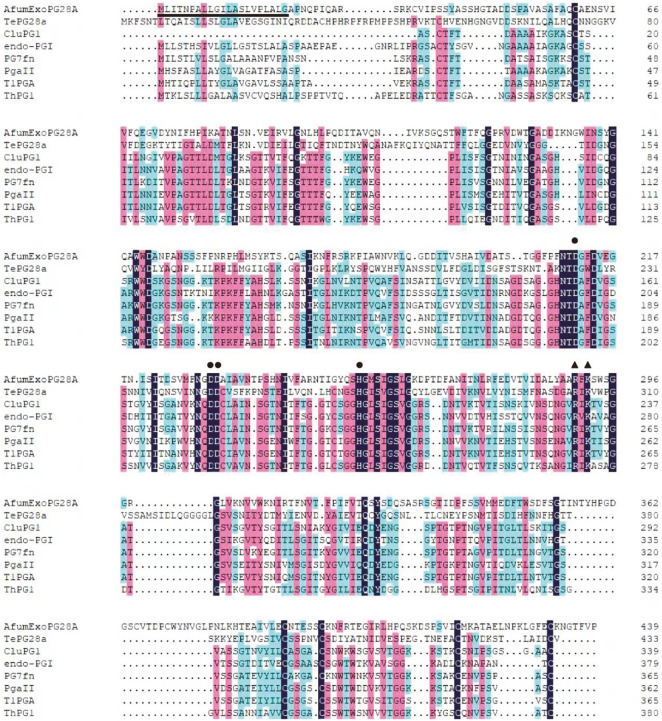
Multiple sequence alignment of AfumExoPG28A with other functionally characterized GH28 proteins. Amino acid sequence alignment of AfumExoPG28A, *Te*PG28a (from *T. leycettanus*., GenBank Accession No. KY474617), CluPG1 (from *C. lupini*., GenBank Accession No. 2IQ7), endo-PGI (from *Penicillium* sp., GenBank Accession No. AEL22832), PG7fn (from *T. arenaria*., GenBank Accession No. AIZ95162), PgaII (from *A. niger*., GenBank Accession No. CAA41694), T1PGA (from *E. leycettana*., GenBank Accession No. QKK82827), and ThPG1 (from *T. lixii*., GenBank Accession No. CAM07141.1), using DNAMAN software. Amino acid residues showing 100%, >75%, and >50% identity are shaded in light black, light red, and light green, respectively. The signal peptide of AfumExoPG28A is underlined. Asp211, Asp231, Asp232, and His255 of AfumExoPG28A, which were expected to be the catalytic residues, are marked by solid circles. The Arg290 and Lys292 of AfumExoPG28A, which were expected to be involved in substrate binding, are marked by solid regular triangles.

The 1,323-bp coding region of a putative exo-PG gene in the genome of *A. fumigatus* Af293 was obtained from the GenBank database. A putative, 21-residue signal peptide (Met1–Gly21) was predicted by SignalP4.1. In comparison with a functionally characterized protein (PgaII) [24], Asp^211^, Asp^231^, Asp^232^, and His^255^ of AfumExoPG28A were expected to be the catalytic residues, and Arg^290^ and Lys^292^ of AfumExoPG28A were expected to be involved in substrate binding (Fig. 1). However, AfumExoPG28A shared the highest homology of only 27% with an experimentally verified exo-PG (*Te*PG28a) from *Talaromyces leycettanus* [25] (GenBank Accession No. KY474617). Thus, AfumExoPG28A was a novel exo-PG, which is worthy of further study.

**Fig. 2:**
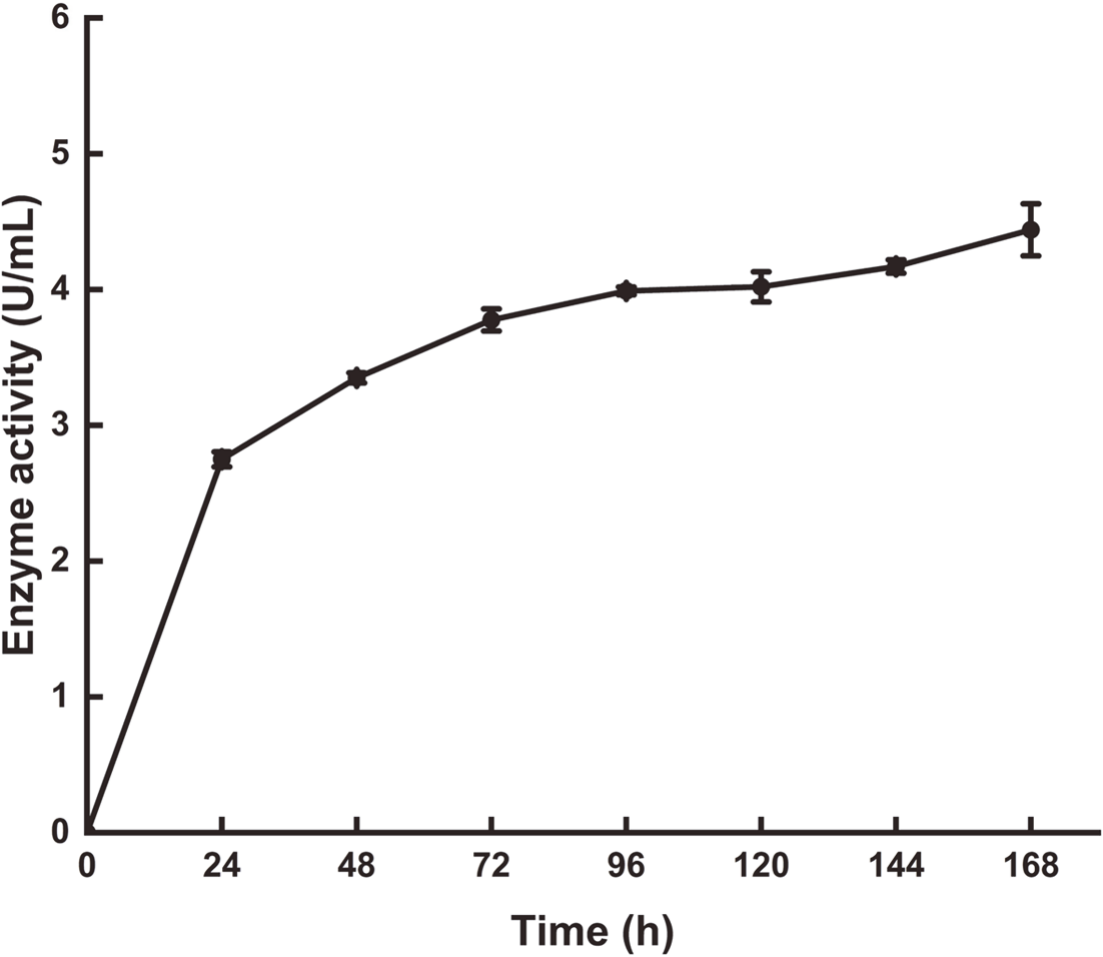
Time course of exo-polygalacturonase production by *P. pastoris*.

The recombinant vector pPIC9K-*AfumExoPG28A* was constructed without a putative signal peptide region and then successfully transformed to *P. pastoris* GS115. After 5 days of cultivation under the induction of methanol, a transformant whose PG activity reached 4.44 U/ml was obtained (Fig. 2). Heterologous expression is an efficient method to obtain novel enzymes. Many PG genes have been cloned and heterologously expressed in eukaryotic and prokaryotic cells with effective expression systems, such as PgaB from *Aspergillus luchuensis* [26, 27], AnEPG from *Aspergillus nidulans* [28], and endoPG from *Aspergillus aculeatus* [29], etc., but only two exo-PG genes (exo-*Te*PG28a from *T. leycettanus* [25] and RmGH28 from *Rhodothermus marinus* [30]) were expressed in *P. pastoris* and *E. coli*, respectively.

**Fig. 3:**
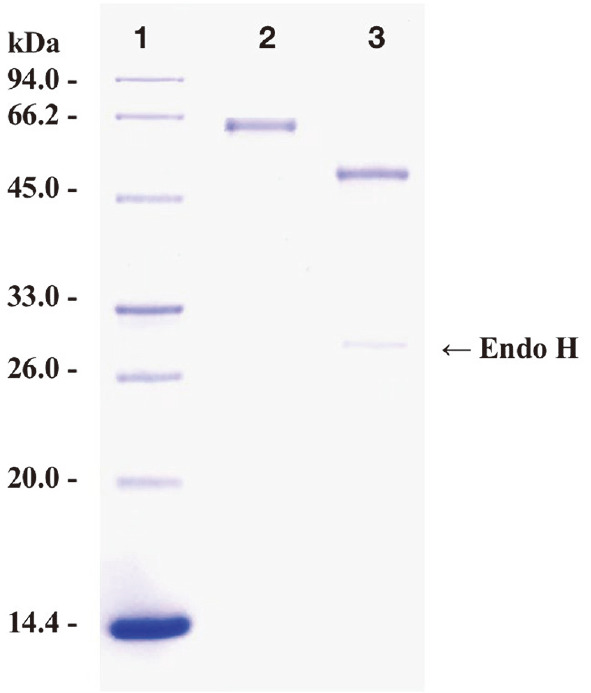
SDS-PAGE analysis of AfumExoPG28A. Lane 1, molecular weight ladder; lane 2, purified recombinant protein; lane 3, purified recombinant protein digested by EndoH.

After a simple one-step procedure of ultrafiltration, the recombinant protein AfumExoPG28A with PG activity was purified to electrophoretic homogeneity, The purified AfumExoPG28A showed a molecular weight of approximately 62 kDa in SDS-PAGE (Fig. 3), which was higher than the calculated value of 46 kDa. After Endo H treatment, the molecular weight of de-glycosylated AfumExoPG28A matched the theoretical value. This confirmed the previously reported observation that heterologous proteins expressed in *P. pastoris* were generally excessively *N*-glycosylated [28, 31].

### Substrate Specificity and Determination of Kinetic Parameters of AfumExoPG28A

**Fig. 4:**
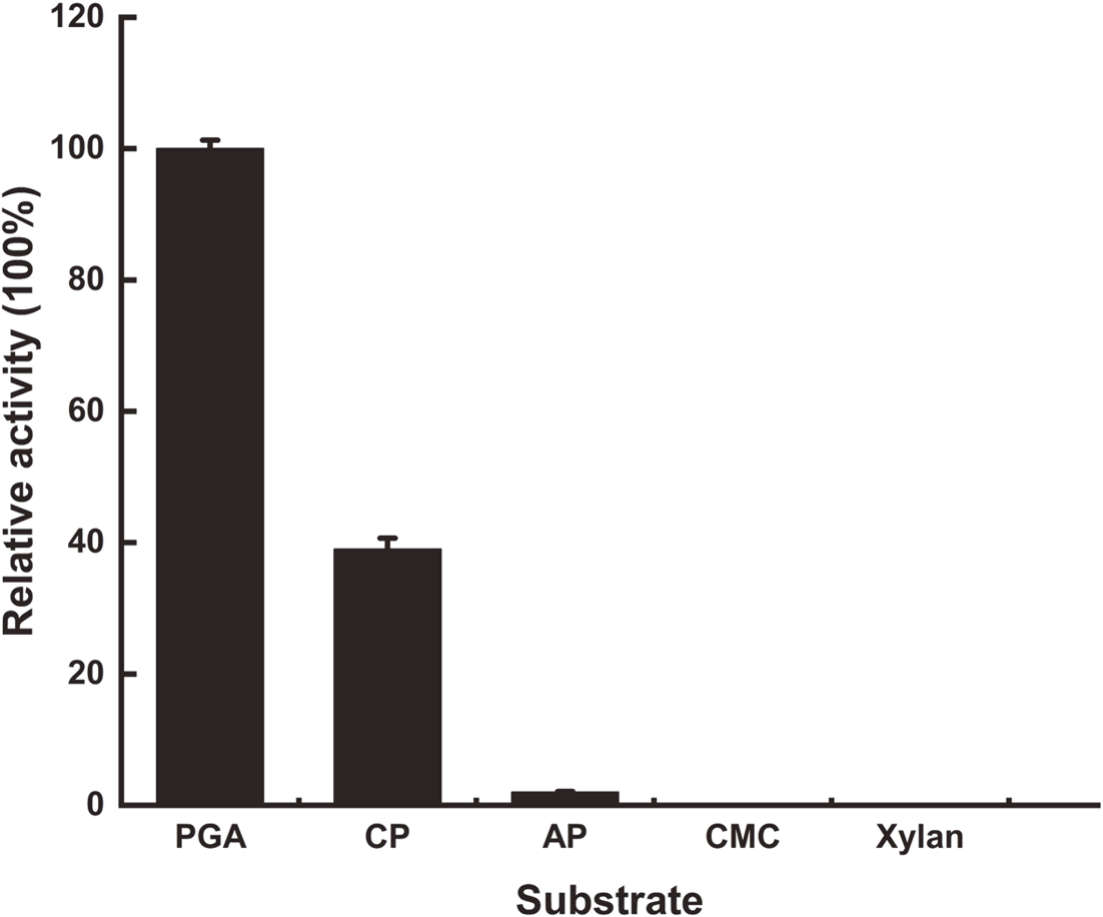
Substrate specificity of AfumExoPG28A. PGA, polygalacturonate acid; CP, low-esterified citrus pectin; AP, high-esterified apple pectin; CMC, carboxymethyl cellulose.

Purified AfumExoPG28A exhibited the highest specific activity towards polygalacturonic acid (PGA) (5.72 U/mg, 100%), followed by citrus pectin (1.44 U/mg, 25%) and apple pectin (0.21 U/mg, 4%), while showing almost no activity on CMC and xylan (Fig. 4). This result agreed with the fact that the methoxy groups can hinder the degradation of the pectin backbone by PGs [32]. Considering the above substrate specificity analysis results, AfumExoPG28A was characterized as a PG.

**Fig. 5:**
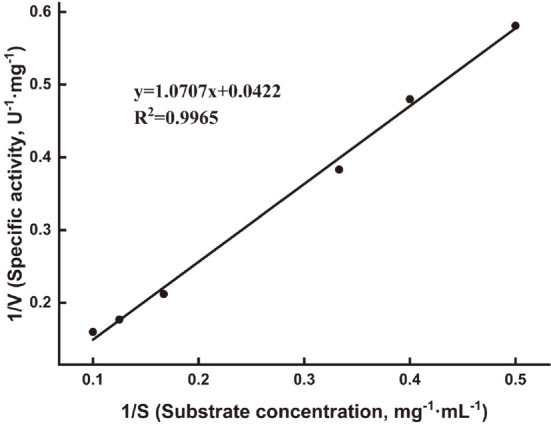
Michaelis-Menten kinetic parameters of AfumExoPG28A. The kinetic parameters of the purified AfumExoPG28A were determined by measuring the reaction rates on different concentrations of PGA ranging from 0.20% to 1.0% under the optimal conditions.

The *K*_m_ and *V*_max_ values of AfumExoPG28A were determined to be 25.4 mg/ml and 23.6 U/mg, respectively (Fig. 5). The *K*_m_ value is significantly higher than those of other PGs from *Neurospora crassa* (5.0 mg/ml) [33], *Paenibacillus amylolytics* (4.6 mg/ml) [34], and *Fusarium moliniforme* (0.12 mg/ml) [35], demonstrating that it may have a lower binding affinity towards PGA than these PGs.

### Effects of Temperature and pH on the Activity and Stability of AfumExoPG28A

**Fig. 6:**
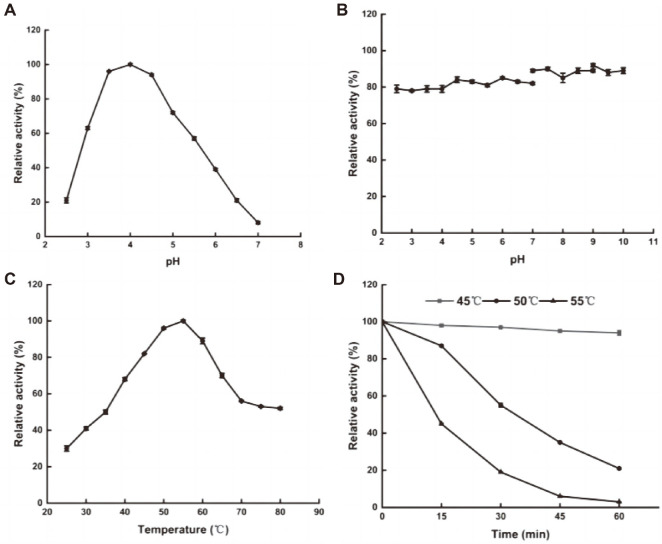
The effects of pH and temperature on the activity of recombinant AfumExoPG28A. (**A**) Effect of pH on recombinant enzyme activity. (**B**) Effect of pH on enzyme stability. (**C**) Effect of temperature on recombinant enzyme activity. (**D**) Effect of temperature on enzyme stability.

The purified AfumExoPG28A showed maximal activity towards PGA at pH 4.0 (Fig. 6A), similar to those of most reported exo-PGs (pH 3.0–5.0) from filamentous fungi [35]. Interestingly, AfumExoPG28A exhibited PG activity over almost the entire acidic pH range (>20.0% activity at pH 2.5–6.5) and remained stable at pH 2.5–10.0 for 24 h (Fig. 6B). PGs with high activity in acidic environments are widely used in fruit juice production and feed processing [2], but different fruit pulps have different pH values. In addition, feed can only be digested after staying in the acidic intestinal environment of animals for a long time [7]. Therefore, it is important to evaluate the pH range in which PGs are stable and active to determine their potential application value. Although most reported exo-PGs show catalytic activity at different pH values, a few remained active and stable under a wide pH range. For example, like AfumExoPG28A, the two exo-PGs from *Penicillium* sp. [36, 37] were active in a wide pH range, but they were only stable at pH 3.0–5.0 and pH 4.5–6.0, respectively. The optimal pH of exo-PG1 was 4.0, similar to that of AfumExoPG28A, but it remained stable only at pH 4.0–5.0 [38]. The optimal pH of exo-PG *Te*PG28a from *T. leycettanus* was also similar to that of AfumExoPG28A, but it had little activity at pH 5.0 [25]. The two exo-PGs from *Aspergillus* sp. remained stable under acidic conditions, but they had almost no activity when the pH was lower than 4.0 [39, 40]. Compared with these enzymes, AfumExoPG28A was both active and stable in a wide pH range, suggesting that it has great potential application value in agriculture and the food industry.

Temperature affects the spatial structure of the enzyme. High or low temperature reduce the catalytic efficiency of the enzyme, and too high temperatures will even cause irreversible protein denaturation [41]. The optimal temperature of AfumExoPG28A was 55°C, similar to most reported optimal temperatures for other PGs [36, 37], and therefore it is a mesophilic pectinase (Fig. 6C). AfumExoPG28A was stable after incubation for 60 min at 45°C, and retained only 30% of its initial activity after incubation for 60 min at 50°C (Fig. 6D). The optimal temperatures of PGs are generally higher than their maximal temperatures of thermostability. The reason may be that higher temperatures facilitate the diffusion of substances, thus increasing the catalytic rate of the enzyme, while the combination of the enzyme and substrate can also improve the stability of the enzyme itself [42].

### The Effects of Metal Ions on AfumExoPG28A Activity

Metal ions affect enzyme activity by binding to amino acid residues. Ca^2+^ serves as an activator of many enzymes, but some studies showed that it could inhibit the activity of PGs [43]. The present study showed that AfumExoPG28A exhibited 73 ± 1.8% enzyme activity in the presence of 2 mM Ca^2+^ (Table 1). Mn^2+^ and Fe^2+^ partially inhibited the activity of AfumExoPG28A, similar to *Penicillium janthinellum* VI2R3M [36]. Cu2+ slightly increased the activity of AfumExoPG28A, similar to *Aspergillus niger* MTCC 478 [39]. The addition of other metal ions had little or no effect on enzyme activity, indicating that the activity of AfumExoPG28A might not depend on the presence of metal ions.

### Analysis of Hydrolysis Products of PGA by AfumExoPG28A

**Fig. 7:**
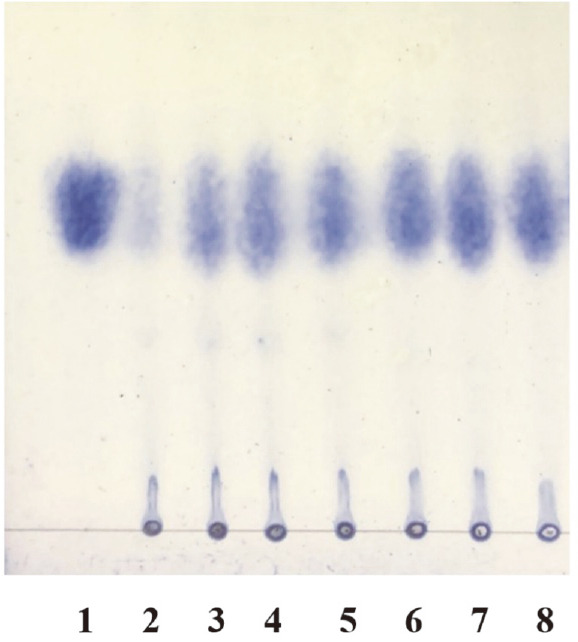
TLC analysis of purified AfumExoPG28A from *A. fumigatus* Af293. Lane 1, galacturonic acid; lanes 2 to 8, liberated products of PGA by AfumExoPG28A for 0, 0.25, 0.5, 1, 2, 8, and 12 h.

The hydrolysis products of PGA by AfumExoPG28A were analyzed by thin-layer chromatography (TLC). As shown in Fig. 7, analysis of products at different time points (0–12 h) showed that no oligosaccharide was produced, and the content of galacturonic acids increased with the extension of reaction time. This behavior was consistent with the previous finding that exo-PG can only hydrolyze the non-reducing terminal glucosyl residues of PGA and produce galacturonic acid monosaccharide products [2], indicating that AfumExoPG28A is an exo-PG.

### Use of AfumExoPG28A for Fresh Plantain Juice Extraction

The cell walls of fruits and vegetables are rich in polysaccharide components such as pectin, cellulose, and hemicellulose, among which pectin can promote the cross-linking of polysaccharide components [2]. Therefore, the addition of pectinase to hydrolyze pectin in juice production can significantly improve the yield and clarity of juice [13]. Endo-PGs show high efficiency of pectin de-polymerization due to their ability to randomly degrade the PGA backbone in pectin molecules; hence, they have attracted wide attention in recent years [28]. While endo-PG is one of the most important pectinases, it had been found that the pectin de-polymerization efficiency could be significantly improved under the synergistic effect of exo-PG and endo-PG [25].

Based on the above results, we identified a novel exo-PG from *A. fumigatus*, indicating that the enzyme could have potential application value in fruit juice production. Next, plantain (*Musa* sp.) was selected as a research object because its pH (pH 4.5) is close to the optimal pH of AfumExoPG28A (pH 4.0). When the enzyme dosage was 0.8 U/mg, AfumExoPG28A increased the light transmittance and yield of plantain juice by 11.7% and 9.0%, respectively. Meanwhile, the endo-PG (0.8 U/mg) from our laboratory increased the light transmittance and yield of plantain juice by 31.1% and 9.0%, respectively. The combinations of AfumExoPG28A and endo-PG increased the light transmittance and yield of plantain juice by 45.7% and 10.0%, respectively (Table 2). In comparison to the AfumExoPG28A or endo-PG alone, the enzyme combinations showed a more significant effect in pectin degradation. This synergy effect may be caused by targeting the terminal groups of the pectic molecule by exo-PG, and randomly cleaving the inner α-1,4 linkages by endo-PG [3]. Therefore, although endo-PG plays a more important role than the exo-PG in pectin hydrolysis, the presence of exo-PG is necessary for complete degradation.

Tropical and subtropical fruits rich in pectin perish quickly, so processing them into juice not only avoids waste but also improves their added value [44]. Some exo-PGs were obtained by purification or heterologous expression, and their application in the processing of tropical and subtropical fruits has also been verified (Table 3). For example, the exo-PG *Te*PG28a from *T. leycettanus* increased the light transmittance of grape juice by 34% [25]; two exo-PGs from *A. niger* [39] and *P. janthinellum* [36] increased the light transmittance of orange juice by 27%and 35%, respectively, and the exo-PG from *Zygoascus hellenicus* [35] increased the light transmittance of tangerine, orange, and grapefruit juices by 3.51%, 4.36%, and 8.04%, respectively. Although the exo-PGs reported above have good potential application value for producing some tropical and subtropical fruit juices, on the whole, the optimal pH of the enzyme was close to the pH value of the fruit pulp, improving the effects of the enzymes.

*A. fumigatus* has a strong capacity for producing glycoside hydrolases that are widely used in the food industry, such as β-D-galactofuranosidases [45], raw starch glucoamylase [46], lipase [47], xyloglucanase [48], and xylanases [49]; however, *A. fumigatus* is an airborne opportunistic pathogen [50, 51]. Thus, cloning and heterologous expression is a highly efficient method for developing and utilizing these enzyme resources.

In this study, a novel exo-PG (AfumExoPG28A) from *A. fumigatus* Af293 was heterologously expressed and characterized. The recombinant protein exhibited the highest activity at 55°C and pH 4.0, while also showing activity and stability in a broad pH range. Moreover, AfumExoPG28A demonstrated excellent performance in juice extraction by increasing the juice yield and light transmittance of plantain pulp. Thus, AfumExoPG28A could be considered as a good candidate enzyme for acidic juice production. This study enriches the existing literature on exo-PGs and provides a theoretical reference for the application of enzymes of this type.

## Figures and Tables

**Table 1:** Effects of metal ions on purified AfumExoPG28A.

Metal ions	Relative activity (100%)	

	1 mM	2 mM
No addition	100.0 ± 1.3	100 ± 0.9
Zn^2+^	96 ± 0.4	103 ± 1.4
K^+^	95 ± 0.4	104 ± 0.3
Ca^2+^	88 ± 0.4	73 ± 1.8
Mg^2+^	102 ± 0.9	99 ± 0.9
Ba^2+^	92 ± 0.8	101 ± 3.1
Na^+^	91 ± 0.6	100 ± 0.5
Mn^2+^	64 ± 0.3	63 ± 0.4
Cu^2+^	119 ± 1.3	122 ± 1.8
Co^2+^	122 ± 0.4	113 ± 0.9
Fe^2+^	95 ± 1.2	87 ± 0.5

**Table 2:** Application of AfumExoPG28A and endo-PG in plantain juice extraction.

Enzyme	%T660	Extraction rate(%)
No addition	41.2 ± 0.2	60 ± 0.1
AfumExoPG28A	52.9 ± 1.2	69 ± 0.2
Endo-PG	72.3 ± 0.3	69 ± 0.9
Combinatorial enzymes	86.9 ± 1.9	70 ± 0.6

**Table 3:** Some biochemical properties of exo-PGs and applications in juice extraction.

Enzyme name	Source strain	Optimal pH and temperature	Fruit	Initial pH of juice	Performance	Reference
AfumExoPG28A	*Aspergillus fumigatus* Af293	4.0, 55°C	Plantain	4.5	ILTa:11.70%, IYb: 9.00%	This study
*Te*PG28a	*Talaromyces leycettanus* JCM 12802	3.5, 70°C	Grape	3.4-3.9	ILTa: 34.00%	[25]
PG	*Aspergillus niger* MTCC 478	4.0, 50°C	Orange	4.1	ILTa: 27.00%	[39]
PG	*Penicillium janthinellum* VI2R3M	5.0, 50°C	Orange	3.5	ILTa: 35.00%	[36]
			Apple	4.0	ILTa: 45.00%	
			Mango	4.5	ILTa: 49.00%	
Exo-PG	*Zygoascus hellenicus* V25	5.0, 60°C	Tangerine	3.94	ILTa: 3.51%	[35]
			Orange	3.47	ILTa: 4.36%	
			Grape	3.58	ILTa: 8.04%	
			Apple	3.82	ILTa: 12.2%	

ILTa: increment of light transmittance (%); IYb: increment of yield (%) .
